# Subjective cognitive functioning in relation to changes in levels of depression and anxiety in youth over 3 months of treatment

**DOI:** 10.1192/bjo.2020.68

**Published:** 2020-08-05

**Authors:** Kelly Allott, Caroline Gao, Sarah E. Hetrick, Kate M. Filia, Jana M. Menssink, Caroline Fisher, Ian B. Hickie, Helen E. Herrman, Debra J. Rickwood, Alexandra G. Parker, Patrick D. Mcgorry, Sue M. Cotton

**Affiliations:** Orygen, Australia; and Centre for Youth Mental Health, The University of Melbourne, Australia; Orygen; Centre for Youth Mental Health, The University of Melbourne; and Department of Epidemiology and Preventive Medicine, School of Public Health and Preventive Medicine, Monash University, Australia; Department of Psychological Medicine, University of Auckland, New Zealand; Orygen; and Centre for Youth Mental Health, The University of Melbourne, Australia; Orygen; and Centre for Youth Mental Health, The University of Melbourne, Australia; Department of Psychology, Royal Melbourne Hospital, Melbourne Health; and The Melbourne Clinic, Australia; Brain and Mind Centre, The University of Sydney, Australia; Orygen; and Centre for Youth Mental Health, The University of Melbourne, Australia; *headspace* National Youth Mental Health Foundation; and Faculty of Health, University of Canberra, Australia; Orygen; Centre for Youth Mental Health, The University of Melbourne; and Institute for Health and Sport, Victoria University, Australia; Orygen; and Centre for Youth Mental Health, The University of Melbourne, Australia; Orygen; and Centre for Youth Mental Health, The University of Melbourne, Australia

**Keywords:** Subjective cognitive functioning, depression, anxiety, youth, longitudinal

## Abstract

**Background:**

Subjective cognitive difficulties are common in mental illness and have a negative impact on role functioning. Little is understood about subjective cognition and the longitudinal relationship with depression and anxiety symptoms in young people.

**Aims:**

To examine the relationship between changes in levels of depression and anxiety and changes in subjective cognitive functioning over 3 months in help-seeking youth.

**Method:**

This was a cohort study of 656 youth aged 12–25 years attending Australian *headspace* primary mental health services. Subjective changes in cognitive functioning (rated as better, same, worse) reported after 3 months of treatment was assessed using the Neuropsychological Symptom Self-Report. Multivariate multinomial logistic regression analysis was conducted to evaluate the impact of baseline levels of and changes in depression (nine-item Patient Health Questionnaire; PHQ9) and anxiety symptoms (seven-item Generalised Anxiety Disorder scale; GAD7) on changes in subjective cognitive function at follow-up while controlling for covariates.

**Results:**

With a one-point reduction in PHQ9 at follow-up, there was an estimated 11–18% increase in ratings of better subjective cognitive functioning at follow-up, relative to stable cognitive functioning. A one-point increase in PHQ9 from baseline to follow-up was associated with 7–14% increase in ratings of worse subjective cognitive functioning over 3 months, relative to stable cognitive functioning. A similar attenuated pattern of findings was observed for the GAD7.

**Conclusions:**

A clear association exists between subjective cognitive functioning outcomes and changes in self-reported severity of affective symptoms in young people over the first 3 months of treatment. Understanding the timing and mechanisms of these associations is needed to tailor treatment.

## Background

Subjective cognitive difficulties, complaints or failures (such as losing one's train of thought, forgetting important information or having trouble concentrating) are commonly experienced by people with mental disorders.^1–3^ Subjective cognitive difficulties are not significantly correlated with objective clinician-administered tests of cognitive performance,^4–9^ but are significantly associated with a range of individual factors, including levels of stress, chronotype, substance use, sleep quality and mental health symptoms to name a few.^2,5,6,10–13^ People with depression and anxiety disorders appear especially susceptible to subjective cognitive difficulties;^1–3,9,14,15^ these difficulties are associated with severity of affective symptoms^16–19^ and have a negative impact on role functioning and quality of life.^9,16,17,20,21^ Younger age is associated with higher levels of subjective cognitive difficulties in people with depression.^3,9,17^ Together, these findings suggest that subjective cognitive functioning is important to consider independently of objective cognition in research, clinical formulation and treatment.

To date, most studies have been cross-sectional and limited to specific diagnostic groups. However, affective symptoms are common to all mental health conditions and little is known about the temporal relationship between subjective cognitive functioning and affective symptoms cross-diagnostically. It is important to understand how subjective cognitive symptoms relate to affective symptoms early in the course of mental illness and over time, given they are shown to be more strongly associated with self-efficacy and functioning than objective cognitive difficulties.^22^ Ongoing subjective cognitive complaints may increase one's risk for maintenance of affective symptoms, relapse or poor functional outcomes.^20,23,24^ Similarly, improvements in subjective cognitive functioning may signal clinical and functional improvement. Subjective cognitive functioning can be easily assessed within clinical settings and thus, research understanding its course and clinical significance remains an important endeavour.

## Aims

The aim of this study was to examine the relationship between changes in levels of depression and anxiety, and changes in subjective cognitive function over the first 3 months of treatment in a large sample of youth aged 12–25 attending primary care mental health services (*headspace*) in Australia. We hypothesised that improvements or worsening in anxiety and depression symptoms over 3 months would be associated with subjective improvements or worsening in cognitive functioning, respectively, while controlling for demographic factors and substance use.

## Method

### Design, setting and participants

This study involved analysis of baseline and 3-month follow-up data from a cohort study examining characteristics and outcomes of young people presenting for mental health treatment: the Comprehensive Outcome Measurement for Youth (Y-COM) study. Participants were recruited from three metropolitan and two regional *headspace* centres across Australia. *headspace* was established in 2006 and is Australia's federally funded primary care service that provides highly accessible evidence-based early intervention and integrated support to people aged 12–25 years experiencing, or at risk of developing, mental or substance use disorders.^25–27^ Centres have a number of health professionals offering services at no or low-cost under the Federal Government's Medicare Benefits Schedule. Each multidisciplinary team has various combinations of general practitioners, psychiatrists, allied health professionals (for example psychologists, social workers, nurses, occupational therapists), vocational specialists, and intake and assessment clinicians comprising an ‘access team’. The *headspace* model typically involves an engagement and assessment session with the Access team to determine the person's presenting concerns and needs. The young person is then referred to the appropriate service provider/clinician(s) for treatment. Young people aged 12–25 years (inclusive) presenting to the study *headspace* centres for the first appointment with concerns about mental health or substance use-related problems were considered eligible for the study. There was no emphasis on self-reported mood or cognitive symptoms for study inclusion.

### Measures

A larger battery of self-report and clinician-rated measures were administered at baseline and follow-up in the primary Y-COM study; only those relevant to the current study are reported here. Demographic characteristics of relevance to this analysis included age and gender assigned at birth. Primary diagnoses based on the DSM-5^28^ were determined by participants’ clinicians.

#### Depression and anxiety

Depressive symptoms were measured using the Patient Health Questionnaire (PHQ9),^29^ a nine-item self-report instrument for measuring depression severity over the previous two weeks. All items are rated on a four-point scale from 0 (not at all) to 3 (nearly every day), providing a 0 to 27 severity score. PHQ9 scores of 5, 10, 15 and 20 were taken as the cut-off points for mild, moderate, moderately severe and severe depression, respectively.^29^ Anxiety symptoms were measured using the Generalised Anxiety Disorder scale (GAD7),^30^ a seven-item self-report instrument for measuring symptoms of anxiety over the previous 2 weeks. All items are rated on a four-point scale from 0 (not at all) to 3 (nearly every day), providing a 0 to 21 severity score. GAD7 scores of 5, 10 and 15 were taken as the cut-off points for mild, moderate and severe anxiety, respectively.^30^

#### Subjective cognitive functioning

Subjective cognitive functioning was assessed at 3-month follow-up using the Neuropsychological Symptom Self-Report (NSSR).^14^ The NSSR is a brief eight-item self-report questionnaire designed to assess an individual's subjective changes in cognitive functioning (for example thinking speed, memory, concentration) since commencing treatment. For each item, on a three-point scale the participant indicates whether their cognitive functioning is (a) better than, (b) the same, or (c) worse than before they started treatment; for the current study, this was over the previous 3 months.

#### Covariates: substance use

Substance use was assessed using the World Health Organization Alcohol, Smoking and Substance Involvement Screening Test (WHO-ASSIST).^31^ In the current study we focused on alcohol and cannabis use as these are the substances most commonly used by young people that may have an impact on cognition.^4^ We defined moderate-to-high alcohol use as a score of 11 and over and moderate-to-high cannabis use as a score of 4 and over in accordance with the WHO-ASSIST guidelines.^31^

### Procedure

Recruitment for the study occurred from September 2016 to April 2018. Research assistants were embedded within the *headspace* Access teams to identify eligible study participants. Advertisements were also placed in the centre waiting rooms to increase visibility of the study among potential participants. Written informed consent was obtained from all participants and a parent/guardian for participants aged <18 years. Following consent, participants completed a battery of self-report measures via tablet computers under the guidance of a research assistant. Participants were contacted 3 months post-baseline to complete the follow-up assessment. Participants were reimbursed AU$30 per assessment (baseline and follow-up).

All procedures contributing to this work complied with the ethical standards of the relevant national and institutional committees on human experimentation and with the Helsinki Declaration of 1975, as revised in 2008. All procedures were reviewed and approved by the University of Melbourne Human Research Ethics Committee, and the local Human Ethics and Advisory Group (1645367.1).

### Statistical analysis

Analyses were conducted using R version 3.6.1. Descriptive statistics such as means (s.d.) and percentages (counts) were used to characterise the cohort. Internal consistency between NSSR items was evaluated using Cronbach's alpha. Multivariate multinomial logistic regression analyses were conducted to evaluate the impact of baseline levels of and changes in anxiety and depression symptoms on different aspects of subjective cognitive function at follow-up, while controlling for other key confounding factors including age, gender, diagnosis, alcohol and cannabis use. Ordinal logistic regression models were not applied because of violation of proportional odds assumption in the data. Because of an issue of multicollinearity between GAD7 and PHQ9, their effects were examined in separate models.

Results from the multinomial logistic regression analyses are presented as relative risk ratios (RRRs) with 95% CIs of subjective improvement (better than before treatment) or decline (worse than before treatment) in cognitive functioning relative to stable (same as before treatment) cognitive functioning that is associated with each unit change in the predictor (for example for each unit increase in PHQ9 score reduction). The level of statistical significance was set at *P* < 0.05 (two-sided). To obtain more accurate estimates, and to control for non-response bias, multiple imputation using chained equations were incorporated in the analysis to address missing data for both the outcome and predictor variables.^32^ Data were imputed for participants who had incomplete data (*n* = 23). Sensitivity analyses were conducted with unimputed data as well as using standardised scores instead of crude scores.

## Results

### Consent and participant flow

**Fig. 1:**
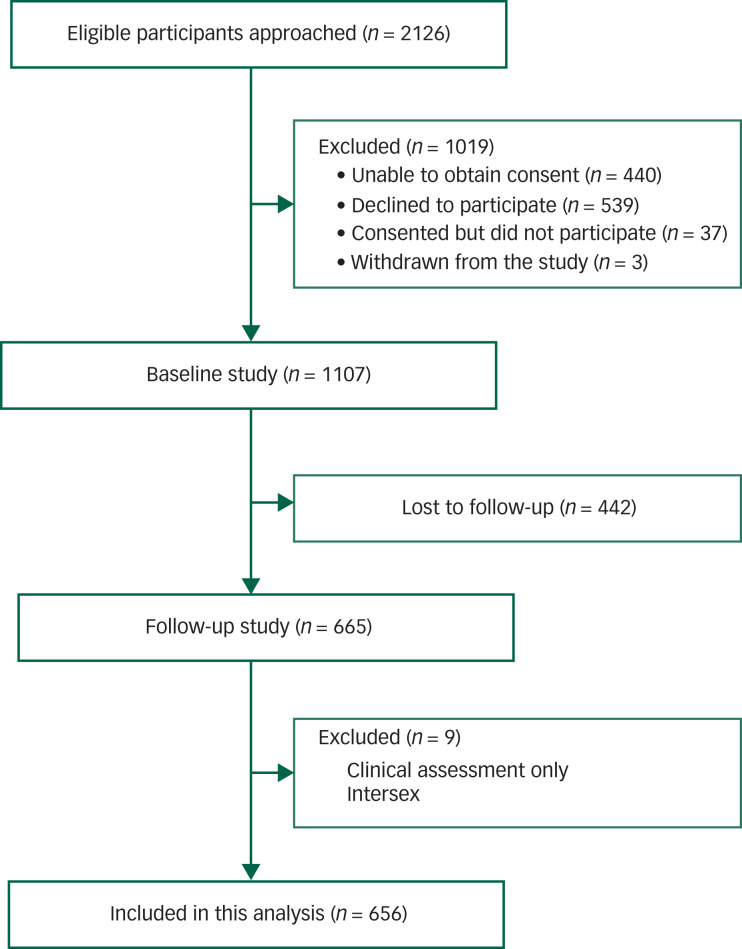
Participant flow diagram.

As shown in Fig. 1, there were 2126 youth across the five *headspace* services who were invited to participate in the study. Of the 1019 youth who were excluded, many declined to participate (52.9%, *n* = 539) or consent could not be obtained because of non-response to being invited or non-attendance at scheduled consenting appointments (43.2%, *n* = 440). In terms of participation, a vast majority of consented (*n* = 1144) participants (96.8%, *n* = 1107) completed the baseline questionnaire battery.

Of those with baseline data, 60.0% (*n* = 665) had follow-up data at 3 months. Of these, a further nine participants were excluded because they did not complete any self-report questionnaires at follow-up or reported being intersex (numbers were too small to be included as a separate group in the analysis). Based on the baseline data, neither demographic characteristics (age and gender) nor clinical factors (diagnosis, anxiety and depression symptoms) were found related to the chance of being lost to follow-up. A total of 656 participants were included in the current analysis.

### Participant characteristics

Table 1 shows that participant age was distributed fairly evenly between the ages of 12 and 25 years, with a slightly lower proportion of participants aged between 12 and 14 years. The mean age of the group was 18.2 (s.d. = 3.3). More participants were female (66.5%). The most frequently found primary diagnoses were anxiety and/or depression (75.9%). A total of 16% of participants reported moderate or high levels of alcohol use and 17% reported moderate or high levels of cannabis use.

**Table 1:** Participants’ characteristics^a^

	Participants (*n* = 656)
Age, mean (s.d.)	18.2 (3.3)
Age group, years: *n* (%)	
12–14	100 (15.2)
15–17	190 (29.0)
18–20	196 (29.9)
21−25	170 (25.9)
Female, *n* (%)	436 (66.5)
Primary diagnosis,^b^ *n* (%)	
Depression	110 (17.4)
Anxiety	182 (28.8)
Depression and Anxiety	188 (29.7)
Other^c^	152 (24.1)
Moderate or high alcohol use, *n* (%)	100 (16.2)
Moderate or high cannabis use, *n* (%)	106 (17.0)

a. Missing data include: 24 participants for primary diagnosis, 40 for alcohol use risk and 34 for cannabis use risk.

b. Primary diagnosis was determined by the participants’ clinicians based on DSM-5.

c. The other diagnoses were as follows: psychosis 2 (0.3%); substance use 9 (1.4%); behavioural disorder 9 (1.4%); personality disorder 19 (3.0%); adjustment disorder 7 (1.1%); provisional diagnosis 15 (2.4%); other 91 (14.4%).

### Depression and anxiety

A substantial proportion of the participants reported high levels of anxiety and depression symptoms. At baseline, the mean PHQ9 score was 12.8 (s.d. = 6.5) and 66.9% had moderate-to-severe depression (see Table 2). The mean PHQ9 symptom score was reduced to 9.8 (s.d. = 6.5) at 3-month follow-up with an average reduction of 3 points (s.d. = 6) and the proportion of participants in the moderate-to-severe depression range was reduced to 46.9%.

**Table 2:** Participants’ anxiety and depression symptoms

	Baseline (*n* = 656)	Follow-up (*n* = 656)
Depression severity measured by PHQ9		
Score, mean (s.d.)	12.8 (6.5)	9.8 (6.5)
PHQ9 risk category, *n* (%)		
Minimal depression (1–4)	79 (12.2)	152 (23.8)
Mild depression (5–9)	136 (20.9)	187 (29.3)
Moderate depression (10–14)	172 (26.5)	138 (21.6)
Moderately severe depression (15–19)	149 (22.9)	107 (16.7)
Severe depression (20–27)	114 (17.5)	55 (8.6)
PHQ9 score reduction from baseline, mean (s.d.)		−3.0 (−6.0)
Anxiety severity measured by GAD7		
Score, mean (s.d.)	10.2 (5.6)	7.9 (5.5)
GAD7 risk category, *n* (%)		
Minimal anxiety (0–4)	115 (17.6)	189 (29.4)
Mild anxiety (5–9)	188 (28.8)	220 (34.3)
Moderate anxiety (10–14)	186 (28.5)	156 (24.3)
Severe anxiety (15–21)	163 (25.0)	77 (12.0)
GAD7 score reduction from baseline, mean (s.d.)		−2.3 (−5.3)
Correlation coefficients		
Between PHQ9 and GAD7	0.67	0.69
Between change of PHQ9 and change of GAD7		0.58

PHQ9, nine-item Patient Health Questionnaire; GAD7, seven-item Generalised Anxiety Disorder scale.

a. Missing data were excluded: 6 records for PHQ9 at baseline, 17 records for PHQ9 at follow-up, 22 records for change of PHQ9 from baseline, 4 records for GAD7 at baseline, 14 records for GAD7 at follow-up and 16 records for change of GAD7 from baseline.

Similarly, the mean GAD7 score at baseline was 10.2 (s.d. = 5.6), which was reduced to a mean score of 7.9 (s.d. = 5.5) at follow-up, with a mean reduction of 2.3 (s.d. = 5.3). The proportion within the range of moderate-to-severe anxiety reduced from 53.5% at baseline to 36.3% at follow-up. Large positive correlations were observed between PHQ9 and GAD7 at both baseline and follow-up (both *r* > 0.60).

### Subjective cognitive function at follow-up

Across each item of the NSSR, most participants reported that their subjective cognition was the same as before starting treatment (see Table 3). About a quarter to a third of participants reported subjective improvement in various aspects of cognitive functioning at 3-month follow-up after treatment. The percentage of participants who reported improvement in their ability to remember verbal instructions and conversations was slightly lower (17.2%) compared with other subjective cognitive symptoms. A small percentage of participants (<10%) reported experiencing subjective deterioration in cognitive functioning since commencing treatment. Internal consistency (Cronbach's alpha based on polychoric correlation coefficients) for the NSSR was 0.93 for the cohort.

**Table 3:** Participants’ Neuropsychological Symptom Self-Report (NSSR) outcomes^a^

	Participants (*n* = 656)
The speed of completing activities	
Worse than before treatment	61 (9.6)
Same as before treatment	407 (64.3)
Better than before treatment	165 (26.1)
Ability to pay attention and concentrate	
Worse than before treatment	58 (9.2)
Same as before treatment	399 (63.0)
Better than before treatment	176 (27.8)
Speed of thinking	
Worse than before treatment	42 (6.6)
Same as before treatment	451 (71.2)
Better than before treatment	140 (22.1)
Ability to remember verbal instructions and conversations	
Worse than before treatment	62 (9.8)
Same as before treatment	462 (73.0)
Better than before treatment	109 (17.2)
Ability to think of words and get words out when speaking	
Worse than before treatment	61 (9.6)
Same as before treatment	400 (63.2)
Better than before treatment	172 (27.2)
Ability to plan ahead and organise things	
Worse than before treatment	37 (5.8)
Same as before treatment	399 (63.0)
Better than before treatment	197 (31.1)
Ability to think about more than one thing at a time	
Worse than before treatment	33 (5.2)
Same as before treatment	432 (68.2)
Better than before treatment	168 (26.5)
Motivation to do my usual activities	
Worse than before treatment	64 (10.1)
Same as before treatment	323 (51.0)
Better than before treatment	246 (38.9)

a. A total of 23 out of 656 participants did not complete the NSSR questions.

### Association between subjective cognitive function and symptoms of anxiety and depression

A clear association was observed between subjective cognitive function outcomes (NSSR) and changes in self-reported severity of anxiety (measured by GAD7) and depression (measured by PHQ9) symptoms (Supplementary Figs 1 and 2 available at https://doi.org/10.1192/bjo.2020.68).

**Fig. 2:**
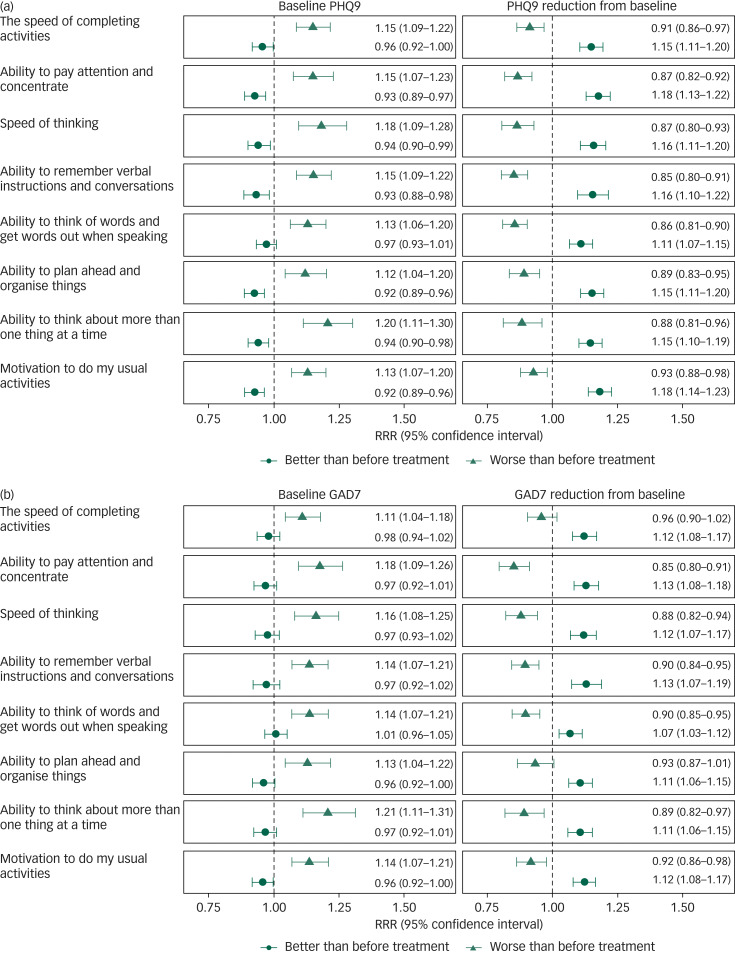
Estimated relative risk ratios (RRR) for better than before treatment and worse than before treatment compared with same as before treatment associated with baseline and reduction from baseline in (a) nine-item Patient Health Questionnaire (PHQ9) and (b) seven-item Generalised Anxiety Disorder scale (GAD7) scores from multiple imputed multinomial logistic regression model controlling for key confounding variables including age, gender, diagnosis, alcohol and cannabis use risk.

Results from multinomial logistic regression models are displayed in Fig. 2. When controlling for baseline PHQ9 scores and other confounding factors, with one-point reduction in PHQ9 from baseline to follow-up, there was an estimated 11–18% increase in rates of reporting better cognitive functioning over the 3 months for different NSSR outcomes. One-point increase of PHQ9 from baseline to follow-up was associated with a 7–14% increase in rates of reporting worsening of cognitive functioning for different NSSR outcomes (see Fig. 2a). Baseline PHQ9 scores were also associated with change in subjective cognitive functioning over 3 months. For those with the same level of change in PHQ9 scores, a higher baseline PHQ9 score was associated with higher chance of deterioration in subjective cognitive functioning, and a lower baseline PHQ9 score was associated with a higher chance of subjective cognitive improvement.

Similar to depression symptoms, associations between baseline and changes in anxiety symptoms and subjective cognitive functioning were also identified. For a one-point reduction in GAD7 scores from baseline to follow-up, there was an estimated 11–13% increased rates of better subjective cognitive functioning for different NSSR outcomes (see Fig. 2b). Increase in GAD7 scores from baseline to follow-up was associated with deterioration of most aspects of subjective cognitive functioning except for ‘ability to plan ahead and organise things’ and ‘the speed of completing activities’.

As with depression, a higher baseline GAD7 score was found to be associated with a higher chance of deterioration in subjective cognitive functioning when controlling for changes in GAD7 scores and other confounding factors. In contrast to depression, there was no evidence of lower baseline GAD7 score being associated with a higher chance of improvement in subjective cognitive functioning. Detailed results from multinomial logistic models are provided in Supplementary Tables 1–8 and models with standardised baseline and change scores of PHQ9 and GAD7 are provided in Supplementary Fig. 3.

## Discussion

### Main findings

To our knowledge this is the first study to examine subjective cognitive functioning in relation to longitudinal change in affective symptoms in youth presenting for mental health treatment. On average, the participants reported moderate levels of depression and anxiety symptoms, which reduced to the mild range over 3 months. Although most participants perceived general stability in their cognitive functioning over the 3-month study period, approximately one-third reported perceived general improvement since commencing treatment. It was concerning, however, that 5–10% of participants perceived a decline in various aspects of their cognitive functioning over the follow-up period.

In support of our hypothesis, the main finding was evidence for a strong association between changes in symptoms of depression and anxiety and changes in subjective cognitive functioning in youth over the first 3 months of mental health treatment. This relationship was the case for every symptom of subjective cognitive functioning assessed when controlling for baseline anxiety and depression symptoms, as well as other key confounders. The estimated effect size associated with changes in symptoms of depression and anxiety were comparable across all cognitive symptoms, which suggests that changes in subjective cognitive functioning in relation to changes in affective symptoms may be a unitary process.

A further finding was that when holding change in symptoms constant, higher or lower baseline levels of depression were significantly associated with perceived decline or improvement in cognitive functioning over 3 months, respectively. For baseline anxiety, higher levels were also associated with a higher chance of deterioration in subjective cognitive functioning, whereas lower baseline anxiety was not associated with a higher chance of improvement in subjective cognitive functioning over 3 months. This suggests that higher severity of affective symptoms at entry to treatment increases one's risk of subjective cognitive decline.

### Comparison with findings from other studies

Our finding of a strong relationship between affective symptoms and subjective cognition are consistent with a large body of cross-sectional research conducted in adult samples with affective disorders (for example ^11,16,17–19^). We extend this work by demonstrating a relationship between affective symptoms and subjective cognitive function in a sample of young people with various mental health concerns and diagnoses, which suggests that the relationship between these variables occurs early in the course of mental illness and is not diagnosis specific. Furthermore, most previous work has focused on depressive symptoms and subjective cognition, but here we have shown that a similar relationship exists with symptoms of anxiety. In support of these findings, previous cross-sectional research both in first-episode psychosis and established schizophrenia showed that depressive and anxiety symptoms were the strongest predictors of subjective cognitive functioning, after accounting for objective cognitive functioning, medication, positive and negative symptoms.^5,33^

### Interpretation of our findings

The findings of the current study support the notion that subjective cognitive impairment is at least in part a state-related phenomenon.^34^ However, because we measured subjective change in cognitive functioning only at the follow-up time point, we were unable to determine what level the participants perceived their cognitive functioning to be at service entry (baseline). This is especially relevant for the largest group who perceived no change in their cognitive functioning over 3 months. It is possible that despite perceived stability, a subgroup of these participants experienced longstanding subjective cognitive impairments. The same could be said for those who perceived improvement in cognitive functioning, where, despite improvement, they still may be performing below their desired level.

Our data do not elucidate the timing of subjective cognitive difficulties and affective symptoms in these young people. Gaining a clearer understanding of the dynamic interplay between these symptoms over time will help inform interventions and clinical recommendations. Temporal relationships may also differ between individuals. For example, subjective cognitive difficulties may be an early manifestation of the clinical expression of anxiety and depressive symptoms, thus interventions designed to identify and address cognitive complaints early may help to promote mental health in young people. Conversely, if anxiety and depressive symptoms tend to precede cognitive concerns, then treatment focused on reducing affective symptoms may be adequate. Future studies should measure subjective cognitive functioning at multiple time points to better characterise the relationship between subjective cognitive functioning and affective symptoms.

The mechanism(s) underpinning the relationship between affective symptoms and subjective cognitive function are not well understood and likely to be multiple and complex. Several pathways may be considered. First, consistent with some of the diagnostic criteria for depression and anxiety disorders, there are items on the self-report measures that ask about cognitive problems. For example, the PHQ9 specifically asks about concentration difficulties. However, this is unlikely to entirely explain the relationship between affective symptoms and subjective cognitive functioning because the NSSR asks about a number of cognitive functions that do not overlap with the PHQ9 or GAD7 and vice versa. Psychological models posit that cognitive biases or maladaptive schemas might contribute to negative appraisal of cognitive function, which may have a further impact on mood, coping strategies and functioning.^11,35,36^

Alternatively, cognitive failures in daily life may increase one's negative affect.^23^ Research has shown that subjective cognitive impairment in mood disorders is associated with poorer socio-occupational functioning,^37,38^ which may in turn have an impact on mood. A vicious cycle may ensue where cognitive difficulties and affective symptoms feed into one another.^23,36^ When subjective cognitive impairment is perceived to have a negative impact on important aspects of daily life, the influence on the development or exacerbation of depression or anxiety symptoms may be greater. It is also possible that depleted cognitive resources are a consequence of poor sleep and mental fatigue, which are common in anxiety and depression.^12^

A further possibility is the depressive realism hypothesis, which suggests that individuals with depression make more realistic and accurate inferences or judgements than healthy persons.^39^ Recent research in fact provides evidence for an underestimation of cognitive ability in people with depression and an overestimation of cognitive ability in healthy controls relative to their objective cognitive performance.^40^ However, the discrepancy between subjective and objective cognitive functioning in individuals with depression may vary as a function of their age and depression severity.^8^ Clearly, more work is needed to understand how these factors relate to one another.

The current study suggests that subjective cognitive functioning (in addition to objective measures) deserves clinical attention, particularly in those who perceive a decline in their cognitive functioning despite having accessed treatment. Ongoing subjective cognitive impairment is commonly found in people who are in partial or full remission from depression.^11^ A 3-year prospective study of adults with depression found that subjectively reported cognitive symptoms were the most prominent symptoms during both the acute and remission phases of depression; participants reported experiencing cognitive problems 44% of the time while in remission from a depressive episode.^41^ Residual self-reported cognitive symptoms have been shown to increase the odds of depression relapse.^24^ Young people with depression report that subjective cognitive difficulties are distressing and interfere with their role functioning (for example study, work, relationships).^23^ In fact, self-reported cognitive difficulties are shown to mediate as much as 25% of the impact of depression on patients’ role functioning.^20^ Therapeutic interventions that aim to reduce subjective cognitive dysfunction in people who are in remission from depression or anxiety could potentially lead to reduced risk of relapse and enhance their functioning. Recent work has shown that providing psychoeducation to young people about cognitive functioning and strategies for managing cognitive difficulties via a fact-sheet was a simple, acceptable and helpful means for beginning to address cognitive concerns in clinical practice.^42^

### Strengths and limitations

There are several strengths of the current study, including the large number of participants and prospective design – previous studies had small sample sizes and were mostly cross-sectional. To our knowledge, no previous study had included youth (adolescents) in their samples. We were also able to control for potential effects of substance use on subjective cognitive functioning in young people presenting for treatment. In previous research examining subjective cognition and affective symptoms there has generally been a lack of consideration for the potential role of substance use, most likely because of the complexity of clinical presentations.

This study also has some limitations. First, objective cognitive functioning was not measured in the current study, which is known to be compromised in youth early in the course of mental health conditions.^43,44^ Evidence consistently shows that there is a low correlation between subjective and objective cognitive impairment.^4–7^ It is possible that subjective and objective cognitive assessments capture different, but equally relevant, aspects of cognition in youth mental health. Longitudinal studies that measure both subjective and objective cognitive functioning are needed to understand the trajectory and unique role that subjective and objective cognitive impairments have in relation to symptom expression and psychological and role functioning.

Second, we did not have a baseline level of subjective cognitive functioning; the NSSR was only administered at one time point and relies on retrospective recall of cognitive functioning, which may be unreliable. Third, data regarding medication use was not collected and medication is known to have an impact on subjective cognitive functioning in varied and complex ways. Although the *headspace* model prioritises evidence-based psychological therapies in the first instance for most young people, who are likely to be presenting with depression and anxiety,^45^ it is likely that a proportion of participants were taking medication during the 3-month follow-up period, which may have affected the findings. Fourth, there may have been relevant differences between participants who were excluded and those who participated in the study, which we were unable to measure.

### Future directions

To conclude, we identified a strong association between affective symptoms and subjective cognitive functioning among help-seeking young people attending primary care youth mental health services. We highlighted the clinical implications of evaluating subjective cognitive functioning and severity of depression and anxiety symptoms at entry to mental health services. Further studies are needed to evaluate the psychometric properties of the NSSR, particularly across a diverse population with different types and severity of mental health conditions. Further work is needed to gain more understanding of the longitudinal associations between subjective and objective cognitive functioning and affective symptoms.

## Data Availability

The data that support the findings of this study are available upon reasonable request and pending additional ethical approval, from the senior author, S.M.C. (sue.cotton@orygen.org.au).

## Supplementary material

For supplementary material accompanying this paper visit http://dx.doi.org/10.1192/bjo.2020.68. click here to view supplementary material
