# Subjective cognitive functioning in relation to changes in levels of depression and anxiety in youth over three months of treatment – CORRIGENDUM

**DOI:** 10.1192/bjo.2020.101

**Published:** 2020-09-14

**Authors:** Kelly Allott, Caroline Gao, Sarah E. Hetrick, Kate M. Filia, Jana M. Menssink, Caroline Fisher, Ian B. Hickie, Helen E. Herrman, Debra J. Rickwood, Alexandra G. Parker, Patrick D. Mcgorry, Sue M. Cotton

**Keywords:** Subjective cognitive functioning, depression, anxiety, youth, longitudinal, corrigendum

It has come to our attention that two of the items for the NSSR were incorrectly labelled in Table 3 and Figure 2 (and in the online supplementary materials).

1. Item 1 should be ‘Ability to stay awake during the day’ instead of ‘The speed of completing activities’.
2. Item 4 should be ‘Memory’ instead of ‘Ability to remember verbal instructions and conversations’.

On page 4 we state that “The percentage of participants who reported improvement in their ability to remember verbal instructions and conversations was slightly lower (17.2%)”. This should say “The percentage of participants who reported improvement in their memory was slightly lower (17.2%)”. Also on page 4 we state that “Increase in GAD7 scores from baseline to follow-up was associated with deterioration of most aspects of subjective cognitive functioning except for ‘ability to plan ahead and organise things’ and ‘the speed of completing activities’. ‘The speed of completing activities’ should be replaced with ‘ability to stay awake during the day’.

These errors have no bearing on the study results or data reported, or on the interpretation of the findings.

We sincerely apologise for the error.
