# Supplementary material for: Subjective cognitive functioning in relation to changes in levels of depression and anxiety in youth over 3 months of treatment
Source: BJPsych Open. 2020 Aug 5;6(5):e84. doi: 10.1192/bjo.2020.68 (PMC7453798; doi:10.1192/bjo.2020.68)
Supplement: Supplementary file 1 [file bjosup.zip › S205647242000068Xsup001.docx]

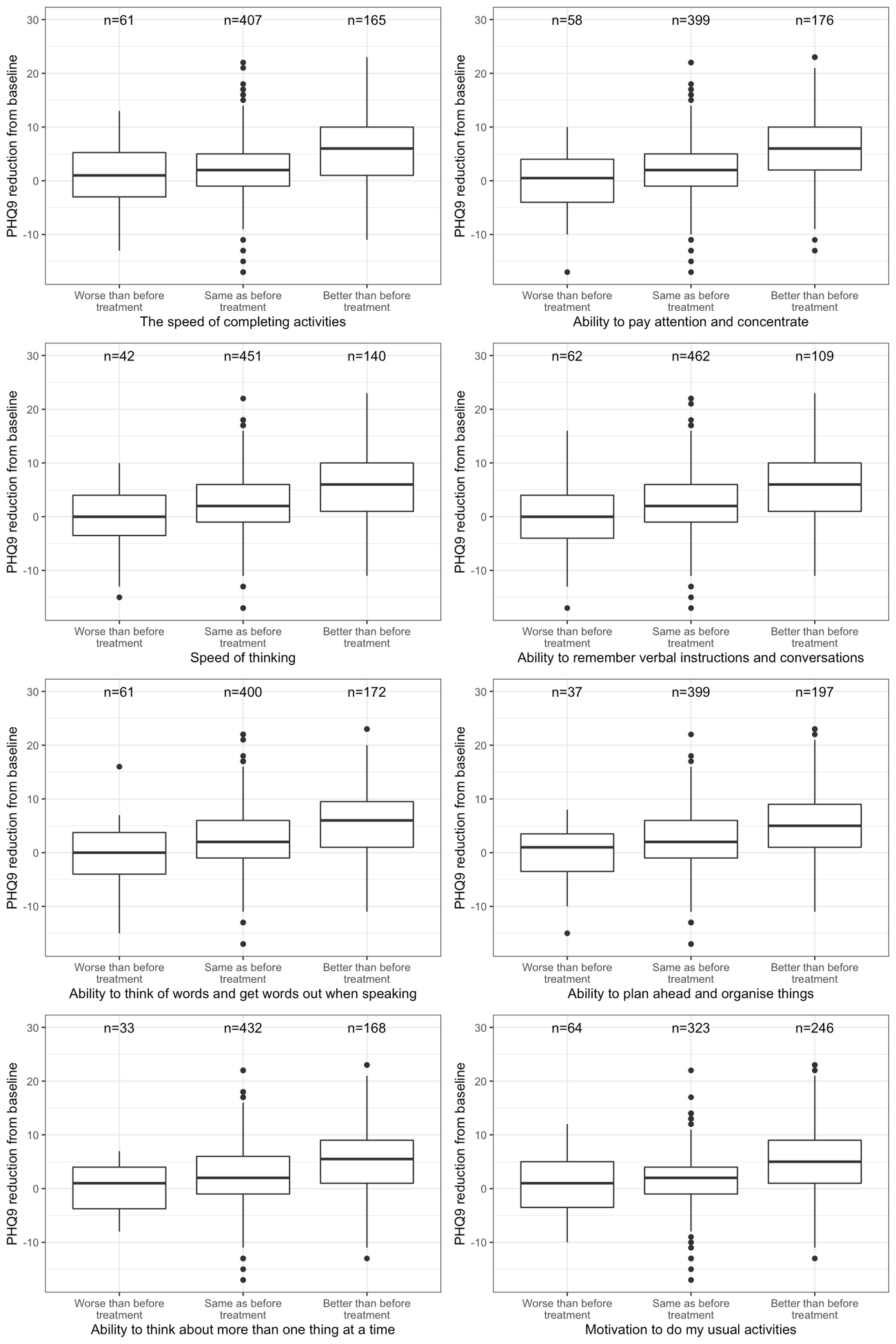


***Supplementary Figure A1.*** Boxplot of changes in PHQ9 from baseline by NSSR outcome groups.


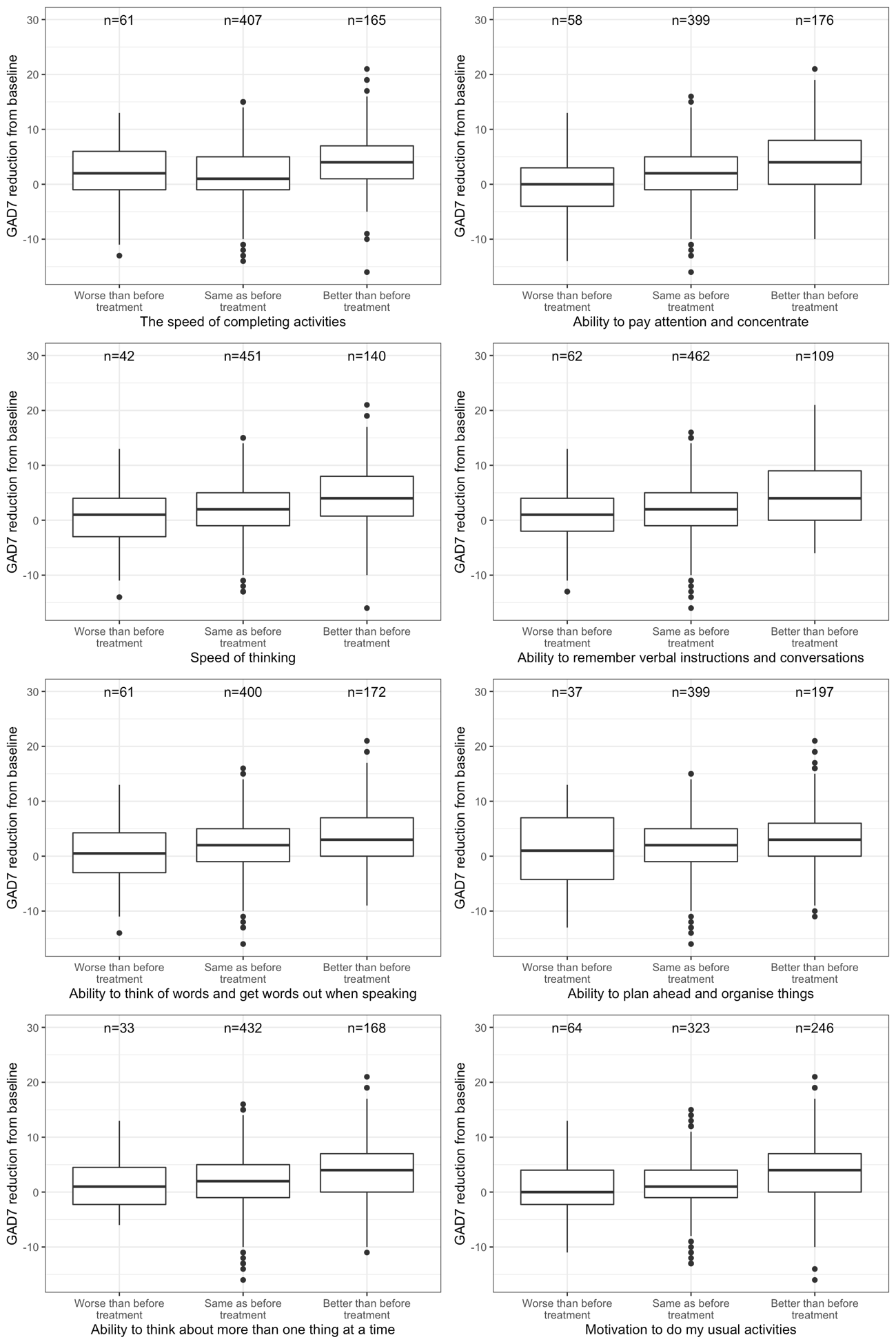


***Supplementary Figure A2.*** Boxplot of changes in GAD7 from baseline by NSSR outcome groups.

Supplementary Table A1. Multinomial logistic regression results for the speed of completing activities

|  | **Model with PHQ9** | | | | | | **Model with GAD7** | | | | | |
| --- | --- | --- | --- | --- | --- | --- | --- | --- | --- | --- | --- | --- |
|  | **Worse than before treatment** | | | **Better than before treatment** | | | **Worse than before treatment** | | | **Better than before treatment** | | |
| **Predictor** | **RRR** | **95% CI** | **p-value** | **RRR** | **95% CI** | **p-value** | **RRR** | **95% CI** | **p-value** | **RRR** | **95% CI** | **p-value** |
| **Score at baseline** | 1.15 | 1.09 - 1.22 | <0.001 | 0.96 | 0.92 - 0.99 | 0.022 | 1.11 | 1.05 - 1.18 | 0.001 | 0.98 | 0.94 - 1.02 | 0.325 |
| **Score reduction from baseline** | 0.91 | 0.86 - 0.97 | 0.002 | 1.15 | 1.10 - 1.20 | <0.001 | 0.96 | 0.90 - 1.02 | 0.182 | 1.12 | 1.07 - 1.17 | <0.001 |
| **Age group** |  |  |  |  |  |  |  |  |  |  |  |  |
| 12-14 | Ref |  |  | Ref |  |  | Ref |  |  | Ref |  |  |
| 15-17 | 4.55 | 1.48 - 13.97 | 0.008 | 0.69 | 0.38 - 1.26 | 0.233 | 4.31 | 1.41 - 13.22 | 0.011 | 0.75 | 0.42 - 1.36 | 0.349 |
| 18-21 | 1.06 | 0.30 - 3.69 | 0.928 | 0.66 | 0.36 - 1.19 | 0.169 | 1.15 | 0.34 - 3.95 | 0.82 | 0.70 | 0.39 - 1.26 | 0.239 |
| 21+ | 1.89 | 0.56 - 6.40 | 0.308 | 0.93 | 0.51 - 1.69 | 0.822 | 1.85 | 0.55 - 6.19 | 0.32 | 1.05 | 0.58 - 1.89 | 0.866 |
| **Sex** |  |  |  |  |  |  |  |  |  |  |  |  |
| Female | Ref |  |  | Ref |  |  | Ref |  |  | Ref |  |  |
| Male | 0.84 | 0.42 - 1.66 | 0.609 | 1.3 | 0.87 - 1.94 | 0.206 | 0.73 | 0.37 - 1.43 | 0.354 | 1.17 | 0.78 - 1.76 | 0.448 |
| **Primary diagnosis** |  |  |  |  |  |  |  |  |  |  |  |  |
| Depression | Ref |  |  | Ref |  |  | Ref |  |  | Ref |  |  |
| Anxiety | 0.61 | 0.22 - 1.67 | 0.340 | 0.96 | 0.48 - 1.89 | 0.899 | 0.39 | 0.15 - 0.99 | 0.047 | 0.99 | 0.55 - 1.81 | 0.984 |
| Depression and Anxiety | 1.24 | 0.52 - 2.94 | 0.626 | 1.3 | 0.68 - 2.48 | 0.423 | 0.95 | 0.42 - 2.15 | 0.905 | 1.21 | 0.67 - 2.19 | 0.520 |
| Other | 1.75 | 0.69 - 4.45 | 0.241 | 1.35 | 0.69 - 2.64 | 0.380 | 1.07 | 0.46 - 2.48 | 0.883 | 1.27 | 0.68 - 2.37 | 0.446 |
| **Alcohol use risk** |  |  |  |  |  |  |  |  |  |  |  |  |
| Low risk | Ref |  |  | Ref |  |  | Ref |  |  | Ref |  |  |
| Moderate or high risk | 1.87 | 0.89 - 3.90 | 0.097 | 0.86 | 0.47 - 1.57 | 0.623 | 1.96 | 0.96 - 4.00 | 0.065 | 0.89 | 0.49 - 1.60 | 0.693 |
| **Cannabis use risk** |  |  |  |  |  |  |  |  |  |  |  |  |
| Low risk | Ref |  |  | Ref |  |  | Ref |  |  | Ref |  |  |
| Moderate or high risk | 0.43 | 0.17 - 1.09 | 0.076 | 0.89 | 0.52 - 1.53 | 0.678 | 0.53 | 0.22 - 1.30 | 0.167 | 0.85 | 0.50 - 1.46 | 0.563 |

Supplementary Table A2. Multinomial logistic regression results for the ability to pay attention and concentrate

|  | **Model with PHQ9** | | | | | | **Model with GAD7** | | | | | |
| --- | --- | --- | --- | --- | --- | --- | --- | --- | --- | --- | --- | --- |
|  | **Worse than before treatment** | | | **Better than before treatment** | | | **Worse than before treatment** | | | **Better than before treatment** | | |
| **Predictor** | **RRR** | **95% CI** | **p-value** | **RRR** | **95% CI** | **p-value** | **RRR** | **95% CI** | **p-value** | **RRR** | **95% CI** | **p-value** |
| **Score at baseline** | 1.15 | 1.08 - 1.22 | <0.001 | 0.93 | 0.89 - 0.96 | <0.001 | 1.18 | 1.10 - 1.26 | <0.001 | 0.97 | 0.93 - 1.01 | 0.097 |
| **Score reduction from baseline** | 0.87 | 0.81 - 0.93 | <0.001 | 1.18 | 1.13 - 1.23 | <0.001 | 0.85 | 0.79 - 0.92 | <0.001 | 1.13 | 1.08 - 1.18 | <0.001 |
| **Age group** |  |  |  |  |  |  |  |  |  |  |  |  |
| 12-14 | Ref |  |  | Ref |  |  | Ref |  |  | Ref |  |  |
| 15-17 | 1.21 | 0.51 - 2.86 | 0.671 | 0.81 | 0.44 - 1.48 | 0.49 | 1.41 | 0.59 - 3.39 | 0.444 | 0.89 | 0.49 - 1.61 | 0.710 |
| 18-21 | 0.33 | 0.12 - 0.90 | 0.03 | 0.75 | 0.40 - 1.37 | 0.348 | 0.4 | 0.15 - 1.11 | 0.078 | 0.84 | 0.46 - 1.55 | 0.581 |
| 21+ | 0.48 | 0.17 - 1.33 | 0.157 | 0.98 | 0.54 - 1.80 | 0.957 | 0.51 | 0.18 - 1.40 | 0.191 | 1.15 | 0.63 - 2.07 | 0.654 |
| **Sex** |  |  |  |  |  |  |  |  |  |  |  |  |
| Female | Ref |  |  | Ref |  |  | Ref |  |  | Ref |  |  |
| Male | 0.61 | 0.30 - 1.27 | 0.189 | 1.14 | 0.76 - 1.70 | 0.525 | 0.69 | 0.33 - 1.45 | 0.326 | 1.09 | 0.73 - 1.62 | 0.685 |
| **Primary diagnosis** |  |  |  |  |  |  |  |  |  |  |  |  |
| Depression | Ref |  |  | Ref |  |  | Ref |  |  | Ref |  |  |
| Anxiety | 0.39 | 0.15 - 1.01 | 0.053 | 1.16 | 0.63 - 2.13 | 0.633 | 0.25 | 0.10 - 0.63 | 0.003 | 1.20 | 0.67 - 2.16 | 0.538 |
| Depression and Anxiety | 0.72 | 0.34 - 1.55 | 0.404 | 1.18 | 0.63 - 2.21 | 0.616 | 0.60 | 0.28 - 1.30 | 0.194 | 1.07 | 0.60 - 1.93 | 0.817 |
| Other | 0.5 | 0.17 - 1.45 | 0.209 | 1.16 | 0.61 - 2.24 | 0.65 | 0.36 | 0.14 - 0.88 | 0.026 | 1.17 | 0.62 - 2.18 | 0.631 |
| **Alcohol use risk** |  |  |  |  |  |  |  |  |  |  |  |  |
| Low risk | Ref |  |  | Ref |  |  | Ref |  |  | Ref |  |  |
| Moderate or high risk | 1.49 | 0.70 - 3.18 | 0.302 | 0.88 | 0.49 - 1.57 | 0.666 | 1.73 | 0.78 - 3.84 | 0.178 | 0.83 | 0.47 - 1.47 | 0.522 |
| **Cannabis use risk** |  |  |  |  |  |  |  |  |  |  |  |  |
| Low risk | Ref |  |  | Ref |  |  | Ref |  |  | Ref |  |  |
| Moderate or high risk | 1.25 | 0.56 - 2.79 | 0.579 | 0.82 | 0.47 - 1.43 | 0.485 | 1.38 | 0.64 - 2.97 | 0.414 | 0.83 | 0.48 - 1.43 | 0.496 |

Supplementary Table A3. Multinomial logistic regression results for speed of thinking

|  | **Model with PHQ9** | | | | | | **Model with GAD7** | | | | | |
| --- | --- | --- | --- | --- | --- | --- | --- | --- | --- | --- | --- | --- |
|  | **Worse than before treatment** | | | **Better than before treatment** | | | **Worse than before treatment** | | | **Better than before treatment** | | |
| Predictor | **RRR** | **95% CI** | **p-value** | **RRR** | **95% CI** | **p-value** | **RRR** | **95% CI** | **p-value** | **RRR** | **95% CI** | **p-value** |
| **Score at baseline** | 1.18 | 1.10 - 1.27 | <0.001 | 0.94 | 0.90 - 0.98 | 0.005 | 1.16 | 1.08 - 1.25 | <0.001 | 0.97 | 0.93 - 1.02 | 0.241 |
| **Score reduction from baseline** | 0.87 | 0.80 - 0.94 | 0.001 | 1.16 | 1.10 - 1.21 | <0.001 | 0.88 | 0.82 - 0.95 | 0.001 | 1.12 | 1.07 - 1.17 | <0.001 |
| **Age group** |  |  |  |  |  |  |  |  |  |  |  |  |
| 12-14 | Ref |  |  | Ref |  |  | Ref |  |  | Ref |  |  |
| 15-17 | 1.68 | 0.56 - 5.00 | 0.354 | 0.61 | 0.33 - 1.12 | 0.112 | 1.64 | 0.56 - 4.79 | 0.364 | 0.64 | 0.35 - 1.17 | 0.144 |
| 18-21 | 1.02 | 0.32 - 3.29 | 0.976 | 0.56 | 0.29 - 1.06 | 0.077 | 1.19 | 0.38 - 3.69 | 0.765 | 0.65 | 0.35 - 1.19 | 0.164 |
| 21+ | 0.66 | 0.19 - 2.37 | 0.527 | 0.64 | 0.34 - 1.21 | 0.172 | 0.70 | 0.20 - 2.44 | 0.572 | 0.76 | 0.41 - 1.41 | 0.387 |
| **Sex** |  |  |  |  |  |  |  |  |  |  |  |  |
| Female | Ref |  |  | Ref |  |  | Ref |  |  | Ref |  |  |
| Male | 2.01 | 0.96 - 4.23 | 0.066 | 1.92 | 1.26 - 2.92 | 0.002 | 1.88 | 0.92 - 3.84 | 0.083 | 1.79 | 1.18 - 2.71 | 0.006 |
| **Primary diagnosis** |  |  |  |  |  |  |  |  |  |  |  |  |
| Depression | Ref |  |  | Ref |  |  | Ref |  |  | Ref |  |  |
| Anxiety | 0.86 | 0.30 - 2.47 | 0.777 | 1.1 | 0.57 - 2.11 | 0.774 | 0.56 | 0.20 - 1.55 | 0.264 | 1.02 | 0.54 - 1.96 | 0.944 |
| Depression and Anxiety | 1.26 | 0.48 - 3.30 | 0.638 | 1.4 | 0.73 - 2.66 | 0.309 | 0.95 | 0.38 - 2.37 | 0.917 | 1.23 | 0.65 - 2.33 | 0.527 |
| Other | 1.19 | 0.41 - 3.40 | 0.752 | 1.44 | 0.75 - 2.77 | 0.276 | 0.88 | 0.32 - 2.36 | 0.793 | 1.35 | 0.70 - 2.59 | 0.365 |
| **Alcohol use risk** |  |  |  |  |  |  |  |  |  |  |  |  |
| Low risk | Ref |  |  | Ref |  |  | Ref |  |  | Ref |  |  |
| Moderate or high risk | 0.88 | 0.36 - 2.20 | 0.792 | 0.86 | 0.46 - 1.58 | 0.621 | 1.06 | 0.44 - 2.54 | 0.905 | 0.87 | 0.48 - 1.57 | 0.636 |
| **Cannabis use risk** |  |  |  |  |  |  |  |  |  |  |  |  |
| Low risk | Ref |  |  | Ref |  |  | Ref |  |  | Ref |  |  |
| Moderate or high risk | 1.34 | 0.57 - 3.15 | 0.502 | 1.15 | 0.65 - 2.05 | 0.635 | 1.41 | 0.62 - 3.19 | 0.411 | 1.12 | 0.64 - 1.94 | 0.692 |

Supplementary Table A4. Multinomial logistic regression results for ability to remember verbal instructions and conversations

|  | **Model with PHQ9** | | | | | | **Model with GAD7** | | | | | |
| --- | --- | --- | --- | --- | --- | --- | --- | --- | --- | --- | --- | --- |
|  | **Worse than before treatment** | | | **Better than before treatment** | | | **Worse than before treatment** | | | **Better than before treatment** | | |
| Predictor | **RRR** | **95% CI** | **p-value** | **RRR** | **95% CI** | **p-value** | **RRR** | **95% CI** | **p-value** | **RRR** | **95% CI** | **p-value** |
| **Score at baseline** | 1.15 | 1.08 - 1.22 | <0.001 | 0.93 | 0.89 - 0.98 | 0.008 | 1.14 | 1.07 - 1.21 | <0.001 | 0.97 | 0.92 - 1.02 | 0.255 |
| **Score reduction from baseline** | 0.85 | 0.81 - 0.90 | <0.001 | 1.16 | 1.10 - 1.22 | <0.001 | 0.90 | 0.84 - 0.95 | <0.001 | 1.13 | 1.07 - 1.19 | <0.001 |
| **Age group** |  |  |  |  |  |  |  |  |  |  |  |  |
| 12-14 | Ref |  |  | Ref |  |  | Ref |  |  | Ref |  |  |
| 15-17 | 0.83 | 0.35 - 1.97 | 0.667 | 0.86 | 0.44 - 1.69 | 0.657 | 0.89 | 0.38 - 2.08 | 0.794 | 0.89 | 0.45 - 1.76 | 0.735 |
| 18-21 | 0.66 | 0.27 - 1.62 | 0.365 | 0.74 | 0.37 - 1.48 | 0.389 | 0.78 | 0.32 - 1.87 | 0.575 | 0.74 | 0.37 - 1.48 | 0.39 |
| 21+ | 0.62 | 0.24 - 1.59 | 0.315 | 0.77 | 0.39 - 1.55 | 0.471 | 0.64 | 0.25 - 1.61 | 0.345 | 0.84 | 0.42 - 1.67 | 0.614 |
| **Sex** |  |  |  |  |  |  |  |  |  |  |  |  |
| Female | Ref |  |  | Ref |  |  | Ref |  |  | Ref |  |  |
| Male | 1.42 | 0.76 - 2.66 | 0.271 | 1.74 | 1.10 - 2.75 | 0.018 | 1.44 | 0.79 - 2.62 | 0.240 | 1.64 | 1.04 - 2.58 | 0.034 |
| **Primary diagnosis** |  |  |  |  |  |  |  |  |  |  |  |  |
| Depression | Ref |  |  | Ref |  |  | Ref |  |  | Ref |  |  |
| Anxiety | 0.93 | 0.38 - 2.26 | 0.869 | 1.07 | 0.50 - 2.26 | 0.866 | 0.59 | 0.26 - 1.34 | 0.211 | 1.00 | 0.49 - 2.04 | 0.997 |
| Depression and Anxiety | 0.94 | 0.42 - 2.09 | 0.876 | 1.5 | 0.73 - 3.08 | 0.267 | 0.73 | 0.34 - 1.58 | 0.427 | 1.25 | 0.62 - 2.52 | 0.535 |
| Other | 0.84 | 0.34 - 2.08 | 0.709 | 1.75 | 0.83 - 3.69 | 0.145 | 0.70 | 0.31 - 1.60 | 0.401 | 1.68 | 0.82 - 3.45 | 0.159 |
| **Alcohol use risk** |  |  |  |  |  |  |  |  |  |  |  |  |
| Low risk | Ref |  |  | Ref |  |  | Ref |  |  | Ref |  |  |
| Moderate or high risk | 1.55 | 0.74 - 3.24 | 0.249 | 0.87 | 0.43 - 1.76 | 0.703 | 1.64 | 0.81 - 3.30 | 0.169 | 0.90 | 0.45 - 1.79 | 0.765 |
| **Cannabis use risk** |  |  |  |  |  |  |  |  |  |  |  |  |
| Low risk | Ref |  |  | Ref |  |  | Ref |  |  | Ref |  |  |
| Moderate or high risk | 1.19 | 0.56 - 2.52 | 0.648 | 1 | 0.54 - 1.87 | 0.999 | 1.28 | 0.64 - 2.57 | 0.482 | 0.99 | 0.52 - 1.88 | 0.965 |

Supplementary Table A5*.* Multinomial logistic regression results for ability to think of words and get words out when speaking

|  | **Model with PHQ9** | | | | | | **Model with GAD7** | | | | | |
| --- | --- | --- | --- | --- | --- | --- | --- | --- | --- | --- | --- | --- |
|  | **Worse than before treatment** | | | **Better than before treatment** | | | **Worse than before treatment** | | | **Better than before treatment** | | |
| Predictor | **RRR** | **95% CI** | **p-value** | **RRR** | **95% CI** | **p-value** | **RRR** | **95% CI** | **p-value** | **RRR** | **95% CI** | **p-value** |
| **Score at baseline** | 1.13 | 1.07 - 1.19 | <0.001 | 0.97 | 0.93 - 1.01 | 0.152 | 1.14 | 1.07 - 1.21 | <0.001 | 1.01 | 0.97 - 1.05 | 0.752 |
| **Score reduction from baseline** | 0.86 | 0.81 - 0.91 | <0.001 | 1.11 | 1.07 - 1.15 | <0.001 | 0.90 | 0.84 - 0.95 | 0.001 | 1.07 | 1.03 - 1.12 | 0.002 |
| **Age group** |  |  |  |  |  |  |  |  |  |  |  |  |
| 12-14 | Ref |  |  | Ref |  |  | Ref |  |  | Ref |  |  |
| 15-17 | 0.55 | 0.23 - 1.31 | 0.178 | 0.6 | 0.32 - 1.10 | 0.101 | 0.59 | 0.26 - 1.38 | 0.226 | 0.61 | 0.34 - 1.11 | 0.109 |
| 18-21 | 0.57 | 0.24 - 1.39 | 0.220 | 0.97 | 0.54 - 1.74 | 0.91 | 0.67 | 0.29 - 1.58 | 0.364 | 1.04 | 0.58 - 1.87 | 0.891 |
| 21+ | 0.57 | 0.23 - 1.42 | 0.230 | 0.84 | 0.46 - 1.55 | 0.578 | 0.55 | 0.22 - 1.34 | 0.188 | 0.94 | 0.52 - 1.69 | 0.832 |
| **Sex** |  |  |  |  |  |  |  |  |  |  |  |  |
| Female | Ref |  |  | Ref |  |  | Ref |  |  | Ref |  |  |
| Male | 1.06 | 0.56 - 2.04 | 0.853 | 1.44 | 0.95 - 2.19 | 0.089 | 1.07 | 0.56 - 2.05 | 0.834 | 1.43 | 0.96 - 2.12 | 0.075 |
| **Primary diagnosis** |  |  |  |  |  |  |  |  |  |  |  |  |
| Depression | Ref |  |  | Ref |  |  | Ref |  |  | Ref |  |  |
| Anxiety | 0.67 | 0.25 - 1.79 | 0.430 | 0.89 | 0.50 - 1.60 | 0.702 | 0.47 | 0.19 - 1.17 | 0.106 | 0.90 | 0.51 - 1.62 | 0.736 |
| Depression and Anxiety | 1.46 | 0.66 - 3.25 | 0.351 | 1.23 | 0.70 - 2.18 | 0.469 | 1.20 | 0.55 - 2.61 | 0.644 | 1.20 | 0.68 - 2.12 | 0.521 |
| Other | 1.02 | 0.40 - 2.62 | 0.96 | 1.06 | 0.56 - 2.00 | 0.857 | 0.77 | 0.32 - 1.85 | 0.563 | 1.07 | 0.58 - 1.97 | 0.827 |
| **Alcohol use risk** |  |  |  |  |  |  |  |  |  |  |  |  |
| Low risk | Ref |  |  | Ref |  |  | Ref |  |  | Ref |  |  |
| Moderate or high risk | 1.08 | 0.50 - 2.36 | 0.843 | 0.84 | 0.47 - 1.48 | 0.546 | 1.17 | 0.55 - 2.49 | 0.684 | 0.80 | 0.47 - 1.38 | 0.430 |
| **Cannabis use risk** |  |  |  |  |  |  |  |  |  |  |  |  |
| Low risk | Ref |  |  | Ref |  |  | Ref |  |  | Ref |  |  |
| Moderate or high risk | 1.05 | 0.48 - 2.29 | 0.908 | 0.97 | 0.57 - 1.63 | 0.904 | 1.10 | 0.52 - 2.30 | 0.808 | 0.96 | 0.57 - 1.62 | 0.884 |

Supplementary Table A6*.* Multinomial logistic regression results for ability to plan ahead and organise things

|  | **Model with PHQ9** | | | | | | **Model with GAD7** | | | | | |
| --- | --- | --- | --- | --- | --- | --- | --- | --- | --- | --- | --- | --- |
|  | **Worse than before treatment** | | | **Better than before treatment** | | | **Worse than before treatment** | | | **Better than before treatment** | | |
| Predictor | **RRR** | **95% CI** | **p-value** | **RRR** | **95% CI** | **p-value** | **RRR** | **95% CI** | **p-value** | **RRR** | **95% CI** | **p-value** |
| **Score at baseline** | 1.12 | 1.05 - 1.20 | 0.001 | 0.92 | 0.89 - 0.96 | <0.001 | 1.13 | 1.05 - 1.22 | 0.002 | 0.96 | 0.92 - 1.00 | 0.054 |
| **Score reduction from baseline** | 0.89 | 0.83 - 0.96 | 0.002 | 1.15 | 1.11 - 1.20 | <0.001 | 0.93 | 0.86 - 1.01 | 0.084 | 1.11 | 1.06 - 1.16 | <0.001 |
| **Age group** |  |  |  |  |  |  |  |  |  |  |  |  |
| 12-14 | Ref |  |  | Ref |  |  | Ref |  |  | Ref |  |  |
| 15-17 | 0.61 | 0.23 - 1.62 | 0.32 | 0.48 | 0.26 - 0.87 | 0.015 | 0.61 | 0.23 - 1.61 | 0.322 | 0.50 | 0.28 - 0.90 | 0.020 |
| 18-21 | 0.49 | 0.17 - 1.43 | 0.192 | 0.79 | 0.44 - 1.40 | 0.413 | 0.5 | 0.17 - 1.42 | 0.193 | 0.83 | 0.47 - 1.46 | 0.519 |
| 21+ | 0.49 | 0.16 - 1.53 | 0.221 | 1.01 | 0.57 - 1.79 | 0.979 | 0.44 | 0.14 - 1.35 | 0.152 | 1.10 | 0.63 - 1.93 | 0.738 |
| **Sex** |  |  |  |  |  |  |  |  |  |  |  |  |
| Female | Ref |  |  | Ref |  |  | Ref |  |  | Ref |  |  |
| Male | 0.94 | 0.43 - 2.08 | 0.88 | 0.84 | 0.56 - 1.26 | 0.406 | 0.91 | 0.41 - 2.02 | 0.821 | 0.84 | 0.57 - 1.25 | 0.387 |
| **Primary diagnosis** |  |  |  |  |  |  |  |  |  |  |  |  |
| Depression | Ref |  |  | Ref |  |  | Ref |  |  | Ref |  |  |
| Anxiety | 1.49 | 0.48 - 4.59 | 0.492 | 1.33 | 0.74 - 2.37 | 0.34 | 1.04 | 0.35 - 3.12 | 0.938 | 1.35 | 0.77 - 2.36 | 0.302 |
| Depression and Anxiety | 0.97 | 0.32 - 2.91 | 0.956 | 1.28 | 0.70 - 2.33 | 0.416 | 0.8 | 0.27 - 2.44 | 0.700 | 1.19 | 0.68 - 2.08 | 0.544 |
| Other | 2.51 | 0.85 - 7.38 | 0.096 | 1.39 | 0.75 - 2.59 | 0.294 | 2.02 | 0.71 - 5.72 | 0.185 | 1.43 | 0.80 - 2.55 | 0.230 |
| **Alcohol use risk** |  |  |  |  |  |  |  |  |  |  |  |  |
| Low risk | Ref |  |  | Ref |  |  | Ref |  |  | Ref |  |  |
| Moderate or high risk | 1.24 | 0.48 - 3.19 | 0.652 | 0.99 | 0.56 - 1.74 | 0.959 | 1.47 | 0.59 - 3.64 | 0.406 | 0.91 | 0.54 - 1.52 | 0.719 |
| **Cannabis use risk** |  |  |  |  |  |  |  |  |  |  |  |  |
| Low risk | Ref |  |  | Ref |  |  | Ref |  |  | Ref |  |  |
| Moderate or high risk | 0.9 | 0.33 - 2.51 | 0.845 | 1.35 | 0.81 - 2.26 | 0.247 | 1.00 | 0.37 - 2.71 | 0.994 | 1.33 | 0.80 - 2.22 | 0.278 |

Supplementary Table A7*.* Multinomial logistic regression results for ability to think about more than one thing at a time

|  | **Model with PHQ9** | | | | | | **Model with GAD7** | | | | | |
| --- | --- | --- | --- | --- | --- | --- | --- | --- | --- | --- | --- | --- |
|  | **Worse than before treatment** | | | **Better than before treatment** | | | **Worse than before treatment** | | | **Better than before treatment** | | |
| Predictor | **RRR** | **95% CI** | **p-value** | **RRR** | **95% CI** | **p-value** | **RRR** | **95% CI** | **p-value** | **RRR** | **95% CI** | **p-value** |
| **Score at baseline** | 1.20 | 1.11 - 1.31 | <0.001 | 0.94 | 0.90 - 0.98 | 0.002 | 1.21 | 1.11 - 1.32 | <0.001 | 0.97 | 0.93 - 1.01 | 0.116 |
| **Score reduction from baseline** | 0.88 | 0.82 - 0.96 | 0.002 | 1.15 | 1.10 - 1.19 | <0.001 | 0.89 | 0.82 - 0.97 | 0.006 | 1.11 | 1.06 - 1.16 | <0.001 |
| **Age group** |  |  |  |  |  |  |  |  |  |  |  |  |
| 12-14 | Ref |  |  | Ref |  |  | Ref |  |  | Ref |  |  |
| 15-17 | 0.96 | 0.32 - 2.85 | 0.944 | 0.59 | 0.33 - 1.05 | 0.075 | 0.91 | 0.31 - 2.62 | 0.855 | 0.62 | 0.35 - 1.10 | 0.104 |
| 18-21 | 0.46 | 0.14 - 1.52 | 0.204 | 0.51 | 0.28 - 0.95 | 0.034 | 0.49 | 0.15 - 1.57 | 0.230 | 0.58 | 0.33 - 1.04 | 0.066 |
| 21+ | 0.28 | 0.07 - 1.09 | 0.067 | 0.66 | 0.36 - 1.20 | 0.176 | 0.31 | 0.08 - 1.20 | 0.092 | 0.73 | 0.41 - 1.30 | 0.283 |
| **Sex** |  |  |  |  |  |  |  |  |  |  |  |  |
| Female | Ref |  |  | Ref |  |  | Ref |  |  | Ref |  |  |
| Male | 2.52 | 1.07 - 5.92 | 0.035 | 1.50 | 1.00 - 2.25 | 0.048 | 2.44 | 1.09 - 5.46 | 0.031 | 1.39 | 0.93 - 2.08 | 0.107 |
| **Primary diagnosis** |  |  |  |  |  |  |  |  |  |  |  |  |
| Depression | Ref |  |  | Ref |  |  | Ref |  |  | Ref |  |  |
| Anxiety | 0.76 | 0.23 - 2.50 | 0.651 | 1.47 | 0.79 - 2.72 | 0.226 | 0.47 | 0.15 - 1.52 | 0.208 | 1.40 | 0.79 - 2.48 | 0.255 |
| Depression and Anxiety | 0.99 | 0.35 - 2.83 | 0.984 | 1.29 | 0.71 - 2.35 | 0.408 | 0.64 | 0.23 - 1.77 | 0.392 | 1.16 | 0.64 - 2.12 | 0.625 |
| Other | 0.89 | 0.25 - 3.15 | 0.862 | 1.07 | 0.57 - 2.03 | 0.833 | 0.59 | 0.19 - 1.82 | 0.362 | 1.02 | 0.55 - 1.88 | 0.946 |
| **Alcohol use risk** |  |  |  |  |  |  |  |  |  |  |  |  |
| Low risk | Ref |  |  | Ref |  |  | Ref |  |  | Ref |  |  |
| Moderate or high risk | 1.19 | 0.40 - 3.51 | 0.751 | 1.07 | 0.61 - 1.88 | 0.814 | 1.32 | 0.51 - 3.43 | 0.567 | 1.06 | 0.60 - 1.85 | 0.848 |
| **Cannabis use risk** |  |  |  |  |  |  |  |  |  |  |  |  |
| Low risk | Ref |  |  | Ref |  |  | Ref |  |  | Ref |  |  |
| Moderate or high risk | 1.73 | 0.66 - 4.53 | 0.263 | 1.09 | 0.64 - 1.85 | 0.758 | 2.02 | 0.81 - 5.01 | 0.131 | 1.13 | 0.67 - 1.91 | 0.645 |

Supplementary Table A8*.* Multinomial logistic regression results for motivation to do my usual activities

|  | **Model with PHQ9** | | | | | | **Model with GAD7** | | | | | |
| --- | --- | --- | --- | --- | --- | --- | --- | --- | --- | --- | --- | --- |
|  | **Worse than before treatment** | | | **Better than before treatment** | | | **Worse than before treatment** | | | **Better than before treatment** | | |
| **Predictor** | **RRR** | **95% CI** | **p-value** | **RRR** | **95% CI** | **p-value** | **RRR** | **95% CI** | **p-value** | **RRR** | **95% CI** | **p-value** |
| **Score at baseline** | 1.13 | 1.07 - 1.20 | <0.001 | 0.92 | 0.89 - 0.96 | <0.001 | 1.14 | 1.07 - 1.21 | <0.001 | 0.96 | 0.92 - 0.99 | 0.021 |
| **Score reduction from baseline** | 0.93 | 0.88 - 0.98 | 0.012 | 1.18 | 1.14 - 1.23 | <0.001 | 0.92 | 0.86 - 0.98 | 0.007 | 1.12 | 1.08 - 1.17 | <0.001 |
| **Age group** |  |  |  |  |  |  |  |  |  |  |  |  |
| 12-14 | Ref |  |  | Ref |  |  | Ref |  |  | Ref |  |  |
| 15-17 | 1.71 | 0.75 - 3.93 | 0.202 | 1.18 | 0.66 - 2.14 | 0.575 | 1.79 | 0.78 - 4.09 | 0.169 | 1.23 | 0.70 - 2.18 | 0.472 |
| 18-21 | 0.60 | 0.23 - 1.53 | 0.281 | 1.39 | 0.77 - 2.49 | 0.273 | 0.7 | 0.27 - 1.79 | 0.453 | 1.5 | 0.85 - 2.65 | 0.166 |
| 21+ | 0.56 | 0.21 - 1.54 | 0.263 | 1.69 | 0.94 - 3.06 | 0.081 | 0.61 | 0.22 - 1.64 | 0.324 | 1.9 | 1.06 - 3.40 | 0.031 |
| **Sex** |  |  |  |  |  |  |  |  |  |  |  |  |
| Female | Ref |  |  | Ref |  |  | Ref |  |  | Ref |  |  |
| Male | 0.65 | 0.33 - 1.30 | 0.221 | 1.12 | 0.76 - 1.63 | 0.574 | 0.61 | 0.30 - 1.25 | 0.178 | 1.08 | 0.74 - 1.57 | 0.688 |
| **Primary diagnosis** |  |  |  |  |  |  |  |  |  |  |  |  |
| Depression | Ref |  |  | Ref |  |  | Ref |  |  | Ref |  |  |
| Anxiety | 0.40 | 0.16 - 1.02 | 0.055 | 1.53 | 0.85 - 2.78 | 0.161 | 0.23 | 0.09 - 0.61 | 0.004 | 1.5 | 0.87 - 2.59 | 0.149 |
| Depression and Anxiety | 0.67 | 0.32 - 1.40 | 0.284 | 1.48 | 0.83 - 2.67 | 0.187 | 0.52 | 0.24 - 1.12 | 0.095 | 1.27 | 0.73 - 2.21 | 0.405 |
| Other | 0.66 | 0.28 - 1.55 | 0.339 | 1.28 | 0.69 - 2.37 | 0.427 | 0.49 | 0.22 - 1.09 | 0.08 | 1.24 | 0.70 - 2.19 | 0.46 |
| **Alcohol use risk** |  |  |  |  |  |  |  |  |  |  |  |  |
| Low risk | Ref |  |  | Ref |  |  | Ref |  |  | Ref |  |  |
| Moderate or high risk | 0.87 | 0.38 - 1.99 | 0.749 | 1.31 | 0.78 - 2.21 | 0.309 | 0.97 | 0.43 - 2.15 | 0.934 | 1.1 | 0.66 - 1.83 | 0.707 |
| **Cannabis use risk** |  |  |  |  |  |  |  |  |  |  |  |  |
| Low risk | Ref |  |  | Ref |  |  | Ref |  |  | Ref |  |  |
| Moderate or high risk | 1.25 | 0.57 - 2.76 | 0.579 | 1.20 | 0.73 - 1.98 | 0.467 | 1.31 | 0.61 - 2.83 | 0.487 | 1.16 | 0.71 - 1.90 | 0.546 |

(A)


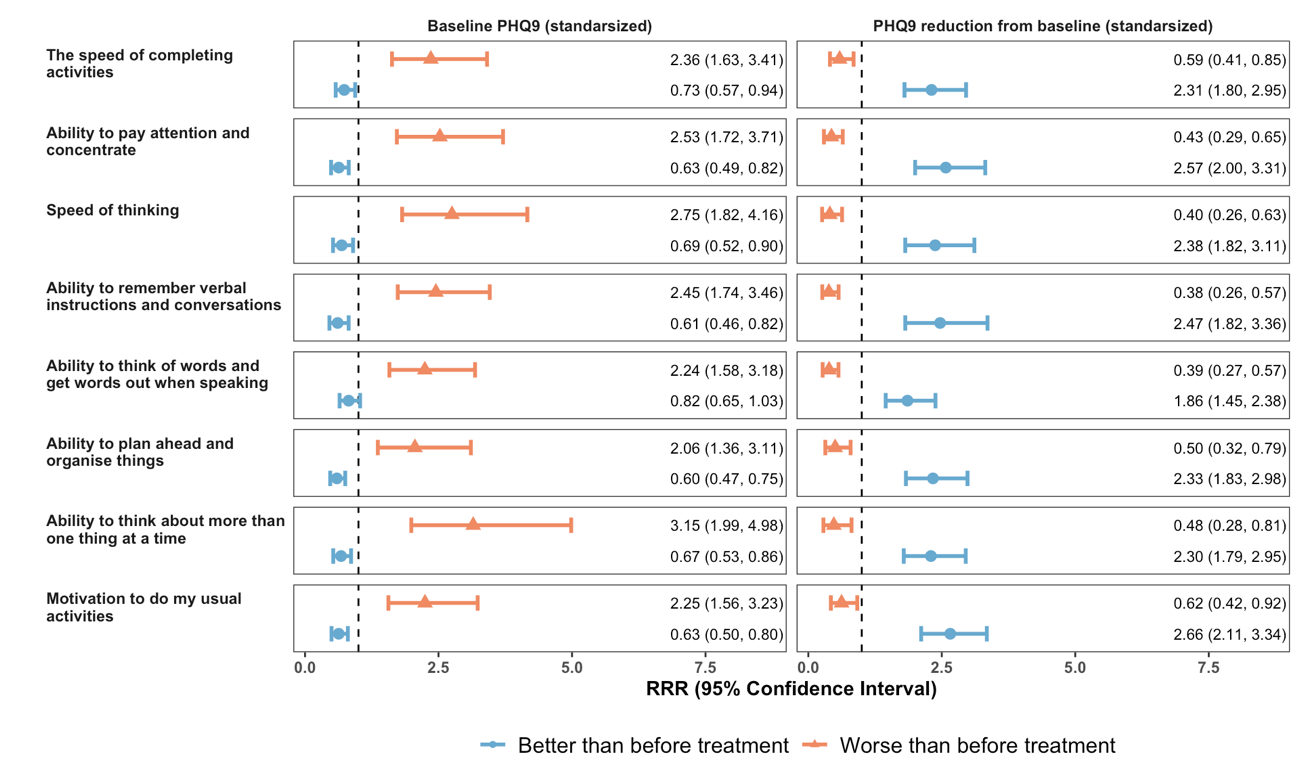


(B)


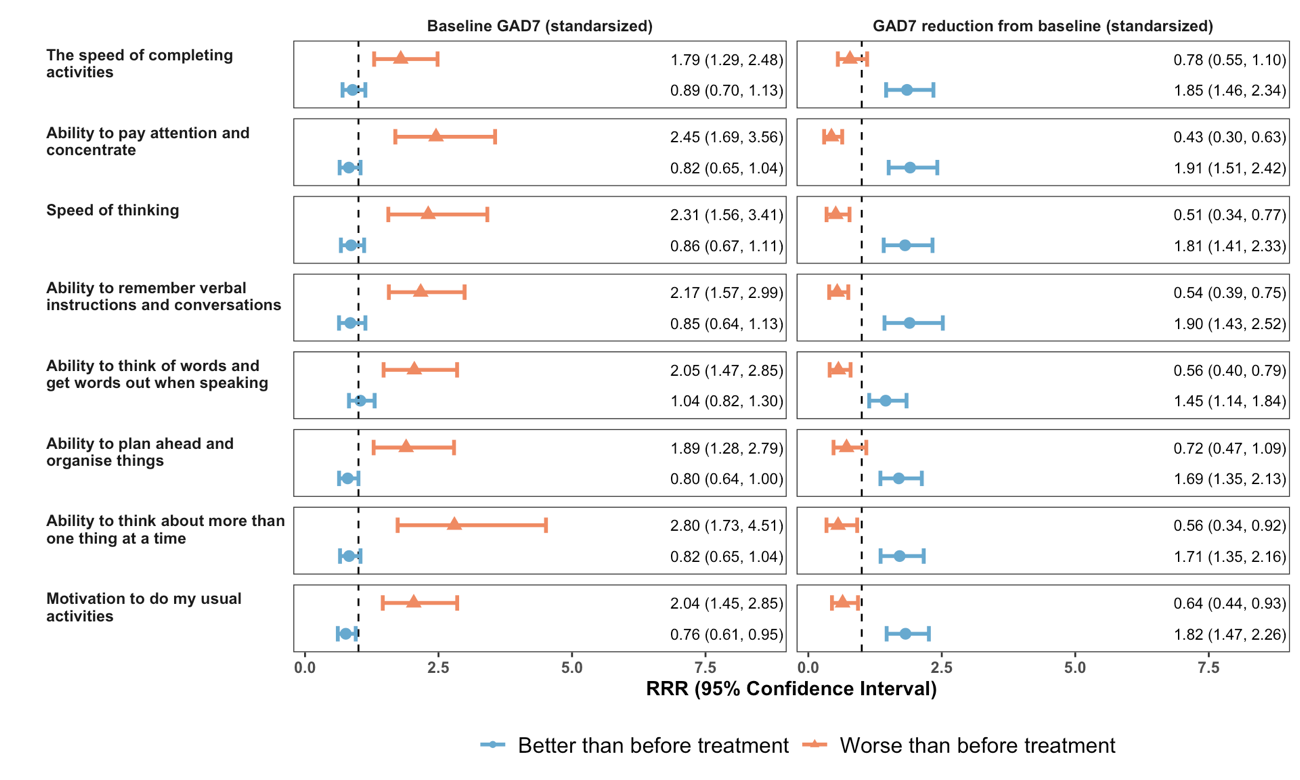


***Supplementary Figure A3.*** Estimated RRR for better than before treatment and worse than before treatment compared with same as before treatment associated with standardized scores of baseline and reduction from baseline in PHQ9 (A) and GAD7 (B) from multiple imputed multinomial logistic regression model controlling for key confounding variables including age, sex, diagnosis, alcohol and cannabis use risk.
